# Exploratory Analysis of Liver Tissue and Preservation Fluid Biomarkers (β-Hydroxybutyrate and Arginase) in Relation to Graft Steatosis

**DOI:** 10.3390/jcm15135239

**Published:** 2026-07-04

**Authors:** Kawthar Safi, Angelika Joanna Pawlicka, Grażyna Kubiak-Tomaszewska, Marta Struga, Andriy Zhylko, Maciej Krasnodębski, Michał Grąt, Alicja Chrzanowska

**Affiliations:** 1 Department of Biochemistry, Medical University of Warsaw, 02-097 Warsaw, Polandalicja.chrzanowska@wum.edu.pl (A.C.); 2 Department of General, Transplant and Liver Surgery, Medical University of Warsaw, Banacha 1A, 02-097 Warsaw, Poland

**Keywords:** liver transplantation, hepatic steatosis, metabolic dysfunction-associated steatotic liver disease, preservation fluid, β-hydroxybutyrate, arginase, extended-criteria donors, metabolic markers

## Abstract

**Background:** Reliable intraoperative tools for donor liver assessment are needed, particularly in the context of steatotic and extended-criteria grafts. While histology remains the reference standard, it is limited by sampling variability and logistical constraints. Preservation fluid may provide a complementary, whole-organ source of biochemical information. **Methods:** In this single-center prospective exploratory pilot study, liver tissue and preservation fluid were collected from 30 donation-after-brain-death grafts during the back-table procedure. Biochemical parameters, including arginase activity, β-hydroxybutyrate (βHB), acetoacetate, and total protein, were measured using standard assays. Associations with histological steatosis on wedge biopsy were assessed using nonparametric correlation analyses, and paired preservation fluid samples were compared. **Results:** Most grafts demonstrated absent or mild steatosis; only two exhibited moderate steatosis, and none were severely steatotic. No preservation fluid biomarker showed a statistically significant association with histological steatosis. Weak, non-significant positive correlations were observed for βHB and arginase activity (Spearman r ≈ 0.33–0.35). Protein concentration and arginase activity decreased between start and end samples, whereas ketone body levels remained relatively stable. **Conclusions:** Preservation fluid biomarker measurement during routine graft preparation is feasible. Although no significant associations with histological steatosis were identified, the observed weak correlations suggest possible associations requiring validation in larger studies. Larger, adequately powered studies, including a broader spectrum of steatosis and clinically relevant outcomes, are required to determine potential clinical applicability.

## 1. Introduction

Liver transplantation (LT) remains the definitive treatment for end-stage liver disease, acute liver failure, and selected liver malignancies. However, the persistent shortage of donor organs continues to limit access and contributes to significant waitlist mortality [1]. To expand the donor pool, increasing use has been made of extended-criteria donor (ECD) grafts, including those with steatosis, advanced donor age, donation after circulatory death, and prolonged ischemia times [2]. Although this approach improves organ availability, such grafts are more susceptible to ischemia–reperfusion injury, early allograft dysfunction, and graft loss, making accurate intraoperative assessment critical for transplant decision-making [3,4,5].

Hepatic steatosis is present in approximately 20–30% of donor livers and remains a major determinant of graft utilization and peri-transplant risk [6]. Steatotic grafts are associated with impaired microcirculation, mitochondrial dysfunction, and increased susceptibility to ischemia–reperfusion injury, particularly in moderate to severe forms [5]. Despite its clinical importance, intraoperative assessment of steatosis remains suboptimal. Macroscopic inspection is rapid but subjective, while histological evaluation, although considered the reference standard, is limited by sampling variability, processing time, and its focal nature [7]. Currently, no simple and routinely applicable biochemical markers in preservation fluid are available for assessing graft steatosis [7].

These limitations are increasingly relevant in the context of metabolic dysfunction-associated steatotic liver disease (MASLD) [8,9]. MASLD is characterized by disordered lipid handling, whereby steatosis represents not only a structural alteration but also a metabolic phenotype characterized by dysregulated lipid metabolism, insulin resistance, oxidative stress, and hepatocellular injury [9,10,11]. As such, structural assessment alone may not fully capture graft vulnerability under ischemic conditions [10].

In this context, preservation fluid obtained during organ procurement and back-table preparation represents a potentially informative and clinically accessible matrix. Biomolecules released into the preservation solution may reflect global graft physiology and biochemical stress in a non-invasive manner. Emerging evidence suggests that perfusate-based biomarkers, including microRNA and glycomic and redox-related signals, may be associated with graft injury and transplant outcomes [12,13,14]. However, many advanced approaches, including machine perfusion and multiomics techniques, remain limited by cost, technical complexity, and availability [4,12]. Additionally, preservation fluid biomarkers have largely been investigated as indicators of graft injury, whereas their ability to reflect the metabolic phenotype associated with graft steatosis remains poorly understood.

**Figure 1:**
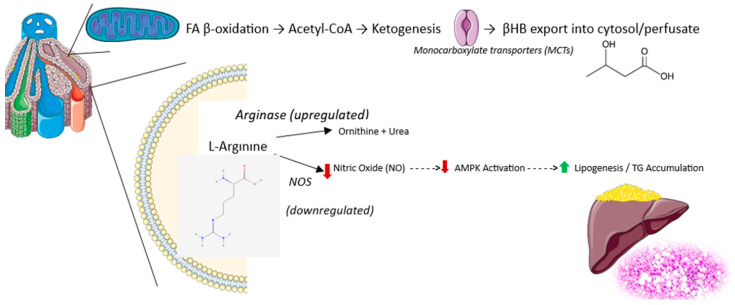
Schematic overview of the metabolic relevance of β-hydroxybutyrate (βHB) and arginase in hepatic steatosis [18,19,20,21]. The diagram illustrates key pathways linking mitochondrial fatty acid oxidation, ketogenesis, and urea cycle activity, and their dysregulation in steatotic liver disease. Solid arrows indicate metabolic conversions, whereas dashed arrows denote regulatory relationships. The illustration was adapted from Servier Medical Art (CC BY 4.0), with additional pathway elements integrated from the cited literature. Red arrows indicated downregulated pathways, and green arrows upregulated pathways.

Given the increasing recognition that steatosis represents not only a structural but also a metabolic phenotype, biomarkers reflecting hepatocellular metabolic function may provide complementary information to conventional injury markers. Among candidate biomarkers, β-hydroxybutyrate (βHB), acetoacetate (AcAc), ketone body ratio (KBR), and arginase are of particular interest because they represent complementary aspects of hepatic metabolism. βHB and AcAc are the principal ketone bodies produced by the liver and reflect mitochondrial fatty acid oxidation and ketogenesis, pathways known to be altered in steatotic liver disease and MASLD [11,15]. The ketone body ratio has been proposed as an indirect indicator of hepatic mitochondrial redox status. Arginase, a highly expressed hepatocellular enzyme, plays a central role in arginine metabolism and the urea cycle. Beyond ammonia detoxification, arginase competes with nitric oxide synthase for L-arginine availability and generates ornithine, linking hepatocellular metabolism with pathways involved in microcirculatory regulation, cellular adaptation, and tissue remodeling [16,17]. Unlike many previously investigated preservation fluid biomarkers that primarily assess graft injury, these markers may provide insight into the metabolic phenotype of donor livers. Figure 1 depicts a schematic overview of the biochemical relevance of β-hydroxybutyrate and arginase in hepatic steatosis, highlighting the link between impaired mitochondrial fatty acid oxidation, altered ketogenesis, and urea cycle activity. These interconnected pathways illustrate how metabolic dysfunction in steatotic livers may lead to measurable changes in β-hydroxybutyrate levels and arginase activity, supporting their evaluation as candidate preservation fluid biomarkers.

In this exploratory pilot study, we evaluated whether β-hydroxybutyrate concentration, arginase activity, and related biochemical parameters (Figure 1) measured in donor liver preservation fluid are associated with histological steatosis in grafts from donation-after-brain-death donors. Grafts with mild and moderate steatosis were studied. Rather than providing statements on diagnostic or predictive utility, the aim of this preliminary work was to assess feasibility and generate hypotheses regarding whether preservation fluid analysis may capture metabolic signals complementary to biopsy-based structural assessment. Given the limited representation of moderate steatosis and the absence of severely steatotic grafts, the study was not designed to establish diagnostic thresholds or clinical utility.

## 2. Materials and Methods

### 2.1. Study Cohort

This single-center prospective study included 30 patients undergoing liver transplantation with donation-after-brain-death (DBD) grafts between April 2024 and March 2025. Ethical approval was obtained for collection of wedge biopsy samples from the left liver lobe. All participants were enrolled according to national transplant regulations and provided informed consent for use of biological material for research purposes.

Liver grafts and preservation fluid samples were pseudonymized using random identifiers. Organs were preserved in cold StoreProtect Plus^®^ (University of Wisconsin–based solution, Carnamedica, Warsaw, Poland) at 4 °C during transport. Upon arrival, grafts underwent standard back-table preparation, including portal vein flushing with 1 L of fresh cold preservation solution. Preservation fluid was collected at the start (S) and end (E) of the back-table procedure (typically lasting 1–4 h). Tissue and fluid samples were stored at −80 °C until analysis. The given study cohort consists of two grafts with moderate steatosis and none with severe steatosis.

### 2.2. Procurement and Graft Implantation

Standard procurement procedures were used with cold in situ perfusion using StoreProtect Plus^®^ solution. Cold ischemia time was recorded for all grafts (mean: 8 h, 13 min). Graft implantation was performed using either the piggy-back or classical technique according to surgeon preference and clinical indication. Reperfusion was initiated via the portal vein followed by arterial reperfusion.

### 2.3. Preservation Fluid Analysis

A panel of established biochemical assays was applied to preservation fluid samples to assess metabolic and injury-related parameters. Preservation fluid samples (see Section 2.1) were analyzed using Exton’s method, the sulphophosphovanillin method, the Chinard method, and the Williamson and Mellanby method with Tomaszewski modification, targeting established biochemical markers of graft metabolism and injury.

These methods were selected on the basis of cost-effectiveness, reproducibility, prior validation in liver studies, and routine use in our laboratory. Absorbance was measured using a ThermoFisher Scientific GENESYS 40/50 UV–Vis spectrophotometer (wavelength accuracy ±0.5 nm, ThermoFisher Scientific, Waltham, MA, USA). All measurements were performed in triplicate.

### 2.4. Biochemical and Tissue Analyses

#### 2.4.1. Total Lipid Concentration (Sulphophosphovanillin Method): Liver Tissue

Lipid content was quantified in tissue by heating samples at 100 °C with concentrated sulfuric acid. Following acid hydrolysis and reaction with phosphovanillin reagent, absorbance was measured at 530 nm.

Each reaction contained 50 µL of sample (50 µL of 1% liver homogenate for tissue assays), 200 µL H_2_SO_4_, while the blank contained 50 µL H_2_O. After vortexing, samples were heated in a boiling water bath for 10 min, cooled, and then mixed with 2 mL phosphovanillin reagent. Following 30 min incubation at room temperature, absorbance was read at 530 nm. Lipid concentration in tissue was determined using a calibration curve (y = 0.001×) generated from cholesterol standards and expressed as percent lipid per gram of tissue. Application of this method to preservation fluid was not feasible due to interference from raffinose in the UW solution, which caused assay artefacts (charring and discoloration under acidic conditions).

#### 2.4.2. Protein/Turbidity (Exton’s Method): Preservation Fluid

Protein-induced turbidity was quantified through precipitation of sample protein in the presence of sulfosalicylic acid and sodium sulfate. Samples consisted of 0.5 mL preservation fluid mixed with 3.5 mL sulfosalicylic reagent (5% sulfosalicylic acid, 16% Na_2_SO_4_). For samples with limited volume, the assay was scaled proportionally.

After vortexing and incubation at room temperature for 10 min, absorbance was read at 445 nm against distilled water. Sample-specific blanks (preservation fluid + saline) were used to correct for baseline turbidity. Protein concentration (mg/L) was calculated from a standard calibration curve (y = 0.002×).

#### 2.4.3. Arginase Activity (Chinard Method): Tissue and Preservation Fluid

Arginase activity was quantified by measuring ornithine production using ninhydrin colorimetric analysis. The assay was applied to both tissue homogenate (µmol/min/g tissue) and preservation fluid (µmol/min/L).

Reaction mixtures contained 0.8 mL carbonate–bicarbonate buffer (pH 10), 0.1 mL sample (preservation fluid or 1% tissue homogenate), followed by heating for 10 min in a boiling water bath. After cooling, 0.1 mL MnCl_2_ (0.01 M) and 0.1 mL L-arginine were added. Samples were incubated at 37 °C for 30 min, then 0.8 mL of 10% trichloroacetic acid (TCA) was added to stop the reaction. After centrifugation (10 min, 4000 rpm), 1 mL supernatant was mixed with 1 mL ninhydrin solution and 3 mL glacial acetic acid and heated for 15 min. Absorbance was read at 515 nm.

Due to high chromogenic intensity, tissue homogenates required 5× to 25× dilution.

Arginase activity was calculated using standard equations. Tissue activity was expressed as µmol/min/g, and preservation fluid activity as µmol/min/L. Units are used consistently throughout the manuscript.

#### 2.4.4. Ketone Bodies (Williamson–Mellanby Method with Tomaszewski Modification): Preservation Fluid

Ketone bodies (acetoacetate [AcAc], β-hydroxybutyrate [βHB], and total ketone bodies [TKB]) were measured in preservation fluid via an enzymatic redox assay based on β-hydroxybutyrate dehydrogenase (HBDH), monitored spectrophotometrically at 340 nm.

Samples and standards were prepared in duplicate. Reaction mixtures contained TRIS/HCl/EDTA buffer (pH 8.5), NAD^+^ (10 mM), and HBDH enzyme (31.36 U/mL). After incubation at room temperature for 15 min, absorbance was measured at 340 nm. Concentrations were calculated relative to an 8 mM AcAc standard and corrected for dilution.

Total ketone bodies were calculated as **TKB = [AcAc] + [βHB]**, and ketone body ratio as **KBR = [AcAc]/[βHB]**.

#### 2.4.5. Liver Density (Immersion Method): Tissue

Liver tissue density (mg/cm^3^) was determined using Archimedes’ principle. Samples were weighed in air (W_air) and then immersed in isotonic saline and reweighed (W_imm). Density was calculated as **Density = W_air/(W_air − W_imm)**. Table 1 summarizes assay application across tissue and preservation fluid specimens.

### 2.5. Statistical Analysis

Non-parametric statistical analyses were performed due to the limited sample size and non-normal data distribution (assessed using the Shapiro–Wilk test and D’Agostino–Pearson test).

Paired comparisons between start (S) and end (E) samples were evaluated using the Wilcoxon signed-rank test. Associations between biochemical parameters and histological steatosis were assessed using Spearman’s rank correlation and reported as Spearman’s rho (ρ).

Data are presented as median (interquartile range, IQR) unless otherwise specified. Statistical significance was defined as *p* < 0.05 (two-sided). Analyses were performed using GraphPad Prism version 8 (GraphPad Software, San Diego, CA, USA).

Given the exploratory nature of the study, no adjustment for multiple comparisons was performed; results should therefore be interpreted as hypothesis generating.

## 3. Results

### 3.1. Cohort Characteristics

Thirty donor liver grafts from donation-after-brain-death donors were included. Histological steatosis was assessed using left-lobe wedge biopsies and was absent or mild in most grafts; only two grafts showed moderate steatosis, and no severe steatosis was observed. Due to incomplete donor and recipient metadata factors such as donor BMI, age, cause of death, and graft outcome were not available. Instead, analyses focused on associations between histological steatosis and biochemical measurements from tissue and preservation fluid. Steatosis grades from histopathological reports refer to macrovesicular steatosis. A detailed breakdown of steatosis grades, in accordance with the NASH CRN (Nonalcoholic Steatohepatitis Clinical Research Network) Kleiner classification, and transplant indications are presented in Table 2 and Table 3.

### 3.2. Descriptive Statistics of Tissue and Preservation Fluid Parameters

Descriptive statistics for liver tissue and preservation fluid parameters are summarized in Table 4. Variables included hepatic steatosis percentage, tissue lipid content, tissue density, arginase activity, and preservation fluid markers (arginase activity, protein concentration, and ketone bodies measured at the start [S] and end [E] of the back-table procedure).

### 3.3. Inferential Analysis and Correlation: Liver Tissue Analyses

**Figure 2:**
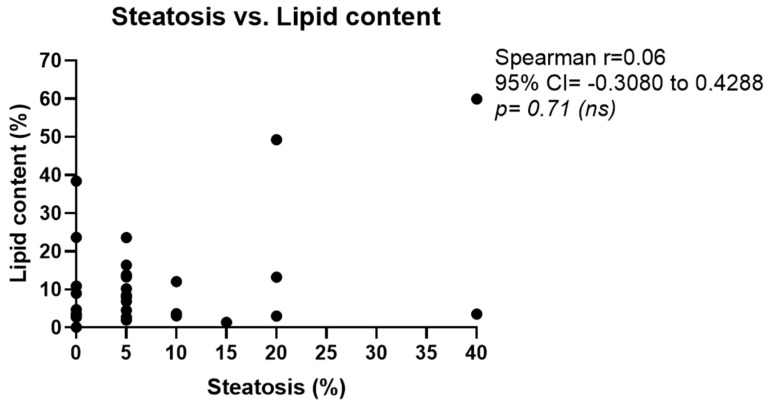
Relationship between steatosis (%) and lipid content (%) across 30 samples. No significant correlation (ns) was found.

**Figure 3:**
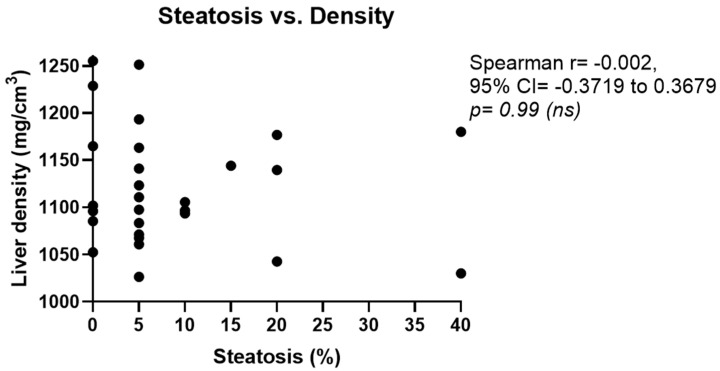
Relationship between steatosis (%) and density across 30 samples. *ns* describes lack of statistical significance.

**Figure 4:**
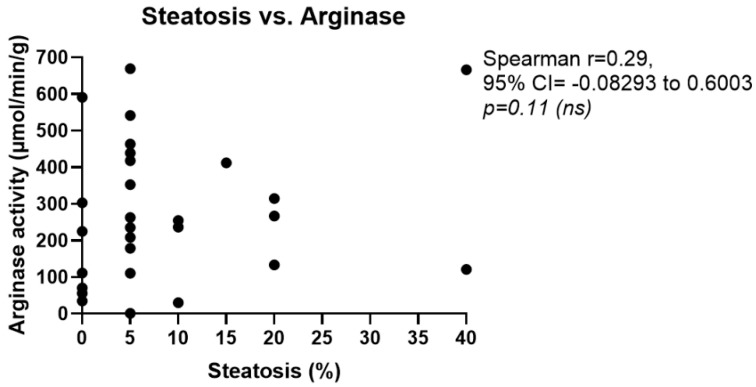
Relationship between steatosis (%) and arginase activity across 30 samples. *ns* describes lack of statistical significance.

Spearman correlation analysis showed no significant association between histological steatosis and tissue lipid content (ρ = 0.06, *p* = 0.71) or liver density (ρ = −0.002, *p* = 0.99). Tissue arginase activity demonstrated a weak positive, non-significant association with steatosis (ρ = 0.29, *p* = 0.11). All confidence intervals included zero. Data trends are visualized in Figure 2, Figure 3 and Figure 4.

### 3.4. Preservation Fluid Correlations

**Figure 5:**
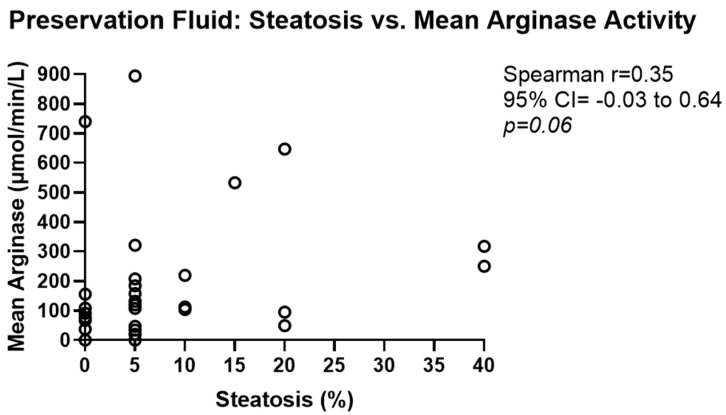
Association between average preservation fluid arginase activity and histological steatosis. Each point represents one graft (*n* = 30). The y-axis shows the mean of start and end arginase activity. Association assessed using Spearman correlation; rho, 95% CI, and exact *p*-value are reported.

**Figure 6:**
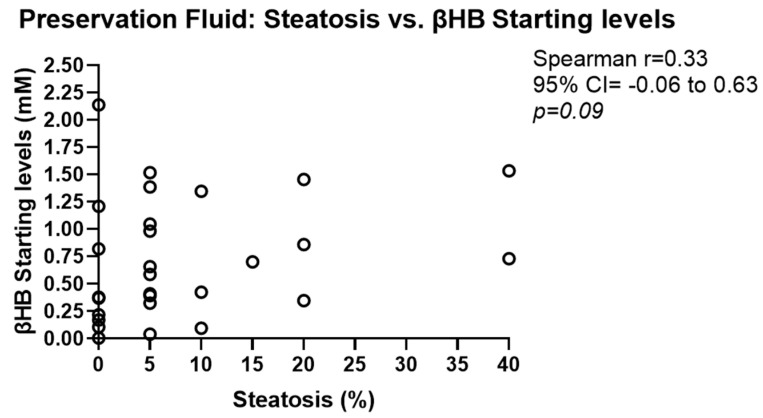
Association between initial preservation fluid β-hydroxybutyrate concentration and histological steatosis. Each point represents one graft (*n* = 30). The x-axis shows steatosis, and the y-axis shows βHB concentration at the start of the back-table procedure. Spearman rho and exact *p*-value are reported.

Spearman correlation analysis demonstrated weak-to-moderate, non-significant associations between histological steatosis and preservation fluid biomarkers (Table 5). Of all the studied parameters, the strongest exploratory associations were observed for mean arginase activity (ρ = 0.35, *p* = 0.060) and β-hydroxybutyrate (ρ = 0.33, *p* = 0.085), displayed in Figure 5 and Figure 6. Despite this, these *p*-values are far from confirmatory and only serve as weak hypothesis-generating signals. No significant correlations were observed for protein concentration, acetoacetate, or ketone body ratio parameters (all *p* > 0.10). Correlation coefficients ranged from −0.27 to 0.35, indicating weak associations overall.

### 3.5. Paired Start vs. End Comparisons

**Figure 7:**
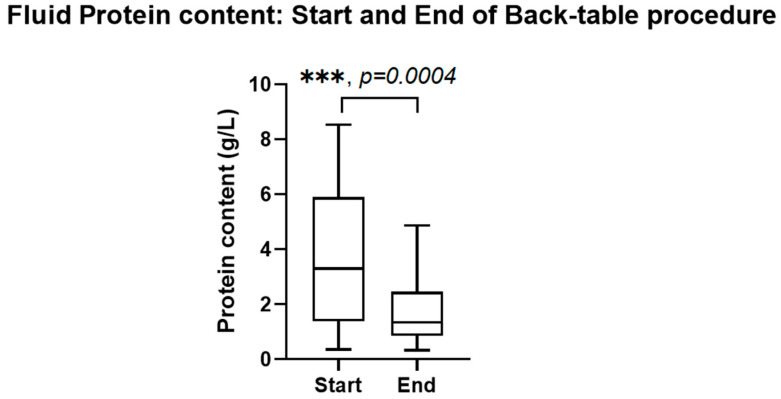
Protein content at the start and end of the back-table procedure. Boxes represent interquartile range (IQR), whiskers indicate min/max, and the line represents the median. Statistically significant difference found by Wilcoxon test, *p* < 0.05 is denoted by ***.

**Figure 8:**
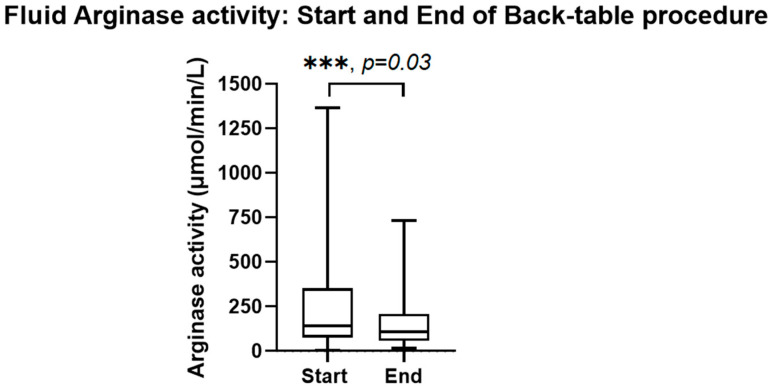
Arginase activity at the start and end of the back-table procedure. Boxes represent interquartile range (IQR), whiskers indicate min/max, and the line represents the median. Statistically significant difference found by Wilcoxon test, *p* < 0.05 is denoted by ***.

Paired comparisons between preservation fluid samples collected at the start and end of the back-table procedure were performed using the Wilcoxon signed-rank test and demonstrated a significant decrease in protein concentration and arginase activity (protein: *p* = 0.004; arginase: *p* = 0.0327). No significant differences were observed for acetoacetate, β-hydroxybutyrate, or ketone body ratio. Results are summarized in Table 6, Figure 7 and Figure 8. For paired analyses, Δ values represent within-graft differences between start and end of back-table perfusate. Paired differences were calculated as end minus start values; negative values indicate a decrease over time.

## 4. Discussion

The increasing use of extended-criteria and marginal liver grafts has intensified the need for objective and practical tools for intraoperative graft assessment. Hepatic steatosis remains a key determinant of graft quality, as it is associated with increased susceptibility to ischemia–reperfusion injury, early allograft dysfunction, and graft loss [22,23,24]. However, current intraoperative assessment relies primarily on macroscopic inspection and histopathological evaluation, both of which are limited by subjectivity, sampling variability, and limited real-time applicability [23].

In this context, preservation fluid represents an accessible and clinically relevant matrix that may provide information beyond focal tissue assessment. Recent studies have increasingly focused on biomarkers reflecting the functional and metabolic status of the liver rather than relying solely on structural assessment. For example, microbiota-based interventions in patients with chronic liver disease have been evaluated using biochemical, inflammatory, and steatosis-related parameters, demonstrating that metabolic alterations may provide clinically relevant information beyond conventional morphological assessment [25]. This concept is consistent with our exploratory approach investigating preservation fluid biomarkers as potential indicators of graft metabolic status. In this exploratory study, β-hydroxybutyrate (βHB) and arginase activity were evaluated as metabolically relevant markers measurable using simple biochemical assays during the back-table procedure. No statistically significant correlations with histological steatosis were identified. Weak-to-moderate, non-significant positive correlations (Spearman r ≈ 0.33–0.35) were observed; however, these findings should be interpreted with caution given the limited sample size and restricted range of steatosis severity. Importantly, the narrow distribution of steatosis within the cohort—predominantly absent or mild, with only two moderately steatotic grafts and no severe cases—likely limited the ability to detect meaningful associations. This represents a key constraint in the present analysis and restricts generalizability to higher-risk graft populations.

Although the present findings remain exploratory, the observed trends may have biological relevance and deserve further consideration. Given the limited sample size and restricted range of steatosis severity, these observations should be interpreted as hypothesis-generating. The weak positive association observed between preservation fluid β-hydroxybutyrate concentration and graft steatosis may reflect alterations in hepatic fatty acid oxidation and ketogenesis [26,27,28,29,30,31,32,33,34,35]. The absence of statistically significant associations is likely related to the limited statistical power of the study rather than the absence of a biological signal.

Unlike ATP or other rapidly fluctuating energy-related metabolites, β-hydroxybutyrate represents a relatively stable end product of hepatic ketogenesis. Because hepatocytes produce but do not significantly utilize ketone bodies, preservation fluid β-hydroxybutyrate may provide an indirect indicator of graft metabolic status. This concept may be particularly relevant in the context of MASLD, where disturbances in lipid handling, mitochondrial function, and ketone body metabolism are well recognized [26,27,28,29,30,31,32,33,34,35].

Arginase activity may also be of biological interest. Although no statistically significant relationship with histological steatosis was identified, the observed trend may reflect alterations in hepatic arginine metabolism. Beyond its role in the urea cycle, arginase regulates L-arginine availability for nitric oxide synthesis and generates ornithine, a precursor for polyamine synthesis and tissue-remodeling pathways. These mechanisms have been implicated in metabolic liver dysfunction and may contribute to the increased vulnerability of steatotic grafts to ischemia–reperfusion injury. Accordingly, arginase activity in preservation fluid may reflect not only hepatocellular enzyme release but also metabolic alterations associated with graft steatosis [16,18,19,20,36,37,38,39,40].

The rationale for investigating β-hydroxybutyrate and arginase differs from that underlying many previously proposed preservation fluid biomarkers. Most reported markers primarily reflect ischemia–reperfusion injury, mitochondrial damage, or inflammatory activation. In contrast, β-hydroxybutyrate and arginase were selected because they are directly linked to key metabolic pathways affected in steatotic and metabolically dysfunctional livers, including ketogenesis, fatty acid oxidation, and arginine metabolism. While the present study did not identify statistically significant associations, the observed trends suggest that preservation fluid biomarkers may capture aspects of both graft injury and the underlying metabolic phenotype, a concept that warrants further investigation in larger cohorts [12,13,30,32,33,34,41,42].

An additional observation is that preservation fluid composition appears to be influenced by procedural factors. The decrease in protein concentration and arginase activity between the beginning and end of the back-table procedure suggests that dilution, flushing, and handling may affect measured analyte levels. Protein concentrations are influenced by multiple variables inherent to organ handling, which limits their metabolic specificity [42]. Such factors include dilution effects, washout of proteins and enzymes during flushing procedures, adsorption to surgical materials, and degradation, thereby influencing measured biomarker concentrations [43]. Moreover, total protein represents a broad mixture of analytes that is influenced by numerous non-biological variables as part of the back-table environment and lacks metabolic specificity compared with targeted metabolites [44]. Preservation fluid measurements may also be influenced by back-table handling, flushing procedures, and dilutional effects, as reflected by changes in protein and arginase levels across the sampling interval. This finding highlights the importance of standardized sampling protocols and suggests that some biomarkers may be more susceptible to ex vivo variability [43,44]. In contrast, ketone body levels appeared relatively stable, which may indicate greater robustness under these conditions.

Previous studies have explored metabolomic and perfusate-based approaches for graft assessment, including lipid profiling and mitochondrial function assays [30,34,41]. While these methods provide detailed biological insights, their clinical application is often limited by technical complexity and resource requirements. In comparison, simple biochemical assays may offer a more accessible exploratory approach, provided that their reproducibility and clinical relevance can be established.

Several limitations must be acknowledged. The study was conducted in a small, single-center cohort and was not powered to detect moderate associations. In addition, the cohort included predominantly non-steatotic grafts, with only a very limited number of moderately steatotic livers, which reflects the lack of availability of suitable research material during the study period. This narrow distribution of steatosis further limits statistical power and reduces the ability to perform robust subgroup analysis across steatosis grades, thereby constraining the interpretability of the observed associations. The restricted spectrum of steatosis further limits interpretability. In addition, the analysis was confined to correlations with histological steatosis and did not include clinically relevant transplant outcomes such as early allograft dysfunction or graft survival. Integration with clinical outcomes will be essential to determine clinical utility. Consequently, no conclusions can be drawn regarding predictive value or clinical utility. Finally, the lack of detailed donor and procedural variables precluded adjustment for potential confounding factors.

Taken together, this study should be considered to be hypothesis generating. It demonstrates the feasibility of measuring metabolically relevant biomarkers in preservation fluid during routine back-table procedures, but does not establish clinically useful markers. Future studies should include larger, well-powered cohorts with a broader range of steatosis severity, standardized sampling protocols, and integration of clinically meaningful outcomes to determine whether preservation fluid biomarkers have practical applicability in liver graft assessment.

## 5. Conclusions

This exploratory pilot study demonstrates the feasibility of measuring β-hydroxybutyrate (βHB) and arginase activity in liver preservation fluid using simple biochemical assays during routine graft preparation. Although no statistically significant associations with histological steatosis were identified, weak non-significant correlations were observed, suggesting potential but unconfirmed links between preservation fluid biomarkers and graft metabolic state. At present, these findings do not support the use of preservation fluid biomarkers for clinical decision making or as a substitute for histopathological assessment.

Future studies should evaluate preservation fluid metabolic markers in relation to clinically relevant post-transplant outcomes, including peak AST and ALT release, early allograft dysfunction (EAD), and composite measures of early graft function such as MEAF score. In addition to evaluating individual biomarkers, future research should investigate integrated approaches combining histological steatosis, cold ischemia time, and preservation fluid metabolic markers. Such analyses may help determine whether composite metabolic–histologic indices better capture overall graft vulnerability and susceptibility to ischemia–reperfusion injury than individual parameters alone. The development and validation of simple composite indices integrating structural, ischemic, and metabolic aspects of graft assessment may represent a promising direction for future research. Larger multicenter cohorts including a broader spectrum of graft steatosis, particularly moderate and severe steatosis, as well as donation-after-circulatory-death (DCD) grafts, will be required to validate these exploratory observations and determine their potential clinical relevance. Furthermore, integration of preservation fluid biomarker assessment with emerging machine perfusion platforms may provide additional opportunities for real-time graft evaluation and viability assessment.

## Figures and Tables

**Table 1:** Summary of assay application.

Assay	Tissue	Preservation Fluid	Notes
Sulphophosphovanillin	Applied	Attempted (not valid)	Raffinose interference in UW solution
Exton	Not applied	Applied	Turbidity-based protein measurement
Chinard	Applied	Applied	Arginase activity (enzyme release marker)
Williamson–Mellanby	Not applied	Applied	Ketogenesis markers (βHB, AcAc)
Density	Applied	Not applied	Archimedes’ immersion method

**Table 2:** Distribution of histological steatosis grades in donor liver grafts (*n* = 30).

Grade	Definition	Frequency (*n*)	%
0	<5%	9	30.0
1	5–33%	19	63.3
2	34–66%	2	6.7
3	>66%	0	0.0

**Table 3:** Indications of liver transplantation.

Indication	Frequency (*n* = 30)
ALD (Alcoholic liver disease)	12
Budd–Chiari syndrome	2
HCV (Hepatitis C virus)	4
AIH (Autoimmune hepatitis)	3
Haemochromatosis	2
HCV + HCC (Hepatocellular carcinoma)	1
PSC + PBC	1
Haemochromatosis + HCC	2
Re-transplant: HAT (Hepatic Artery Thrombosis)	1
ALD/MASLD	1
HBV (Hepatitis B Virus)	1

**Table 4:** Descriptive statistics of liver tissue and preservation fluid parameters: Mean, median, standard deviation (SD), standard error of mean (SEM) and range.

Variable	Mean	Median	SD	SEM	Range
**Liver tissue**					
Steatosis (%)	8.167	5.000	10.540	1.925	40.000
Lipids (%)	11.980	7.378	14.250	2.602	59.820
Density (mg/cm^3^)	1117.000	1100.000	62.120	11.340	228.700
Arginase activity (µmol/min/g tissue)	260.500	235.700	193.500	35.340	669.300
**Preservation fluid**					
Arginase S (µmol/min/L)	258.800	142.400	309.000	58.390	1360.000
Arginase E (µmol/min/L)	162.100	108.400	168.600	31.860	715.300
Protein S (g/L)	3.902	3.299	2.648	0.501	8.184
Protein E (g/L)	1.695	1.336	1.154	0.218	4.545
AcAc S (mM)	0.756	0.575	0.543	0.101	1.801
βHB S (mM)	0.571	0.443	0.451	0.085	1.972
KBR S	2.371	1.173	2.840	0.537	11.310
AcAc E (mM)	0.709	0.586	0.549	0.102	2.133
βHB E (mM)	0.783	0.536	0.664	0.125	2.444
KBR E	1.406	0.788	1.337	0.253	5.068

**Table 5:** Spearman correlation between steatosis and preservation fluid biomarkers.

Comparison	Spearman r	*p*-Value	95% CI	Interpretation
**Steatosis vs. Arginase S**	0.227	0.246	−0.171 to 0.561	Weak positive, not significant
**Steatosis vs. Arginase E**	0.321	0.095	−0.070 to 0.627	Weak–moderate positive, not significant
**Steatosis vs. Avg. Arginase**	0.348	0.060	−0.026 to 0.636	Weak–moderate positive, not significant
**Steatosis vs. Protein S**	0.049	0.806	−0.341 to 0.424	No correlation
**Steatosis vs. Protein E**	−0.143	0.467	−0.499 to 0.254	Weak negative, not significant
**Steatosis vs. Avg. Protein**	0.030	0.877	−0.344 to 0.395	No correlation
**Steatosis vs. AcAc S**	−0.154	0.425	−0.501 to 0.236	Weak negative, not significant
**Steatosis vs. AcAc E**	0.302	0.111	−0.084 to 0.609	Weak positive, not significant
**Steatosis vs. Avg. AcAc**	0.049	0.798	−0.327 to 0.411	No correlation
**Steatosis vs. βHB S**	0.331	0.085	−0.059 to 0.634	Weak–moderate positive, not significant
**Steatosis vs. βHB E**	−0.001	0.995	−0.384 to 0.382	No correlation
**Steatosis vs. Avg. βHB**	0.135	0.479	−0.248 to 0.481	Weak positive, not significant
**Steatosis vs. KBR S**	−0.273	0.160	−0.594 to 0.123	Weak negative, not significant
**Steatosis vs. KBR E**	0.302	0.118	−0.091 to 0.614	Weak positive, not significant
**Steatosis vs. Avg. KBR**	−0.049	0.796	−0.412 to 0.327	No correlation

**Table 6:** Wilcoxon test comparisons: start vs. end of back-table procedure.

Comparison	Δ Median (End − Start)	*p*-Value	Significance
**Arginase S vs. Arginase E (µmol/min/L)**	−0.039	0.032	*
**Protein S vs. Protein E (g/L)**	−1.992	<0.001	*
**AcAc S vs. AcAc E (mM)**	0.132	0.655	**ns**
**βHB S vs. βHB E (mM)**	0.131	0.074	**ns**
**KBR S vs. KBR E**	−0.198	0.171	**ns**

* denotes statistical significance whereas ns the lack of it.

## Data Availability

The original contributions presented in this study are included in the article. Further inquiries can be directed to the corresponding authors.
